# Underlying Genetics of aHUS: Which Connection with Outcome and Treatment Discontinuation?

**DOI:** 10.3390/ijms241914496

**Published:** 2023-09-24

**Authors:** Andrea Spasiano, Daniela Palazzetti, Lucrezia Dimartino, Francesca Bruno, Rocco Baccaro, Francesco Pesce, Giuseppe Grandaliano

**Affiliations:** 1 Department of Translational Medicine and Surgery, Università Cattolica del Sacro Cuore, 00168 Rome, Italy; andrea.spasiano01@icatt.it (A.S.); giuseppe.grandaliano@unicatt.it (G.G.); 2 Nephrology Unit, Department of Medical and Surgical Sciences, Fondazione Policlinico Universitario A. Gemelli, 00168 Rome, Italy; 3 Division of Renal Medicine, Fatebenefratelli Isola Tiberina—Gemelli Isola, 00186 Rome, Italy

**Keywords:** atypical hemolytic uremic syndrome, genetics, aHUS, CFH, MCP

## Abstract

Atypical hemolytic uremic syndrome (aHUS) is a rare disease caused by a genetic dysregulation of the alternative complement pathway, characterized by thrombocytopenia, hemolytic anemia, and acute kidney injury, and included in the group of thrombotic microangiopathies. With the introduction of humanized monoclonal antibodies that inhibit C5 activation, the natural history of aHUS completely changed, with a better prognosis, a quick recovery of renal function, and a significant reduction of end-stage renal disease incidence. Nowadays, there is an increasing interest in the molecular and genetic bases of this severe disease. The aim of this narrative review is to provide readers with a practical guide about different possible involved genes, elucidating the specific role of each transcribed protein in the pathogenesis of aHUS. Moreover, we analyzed the main current evidence about the relationship among genetic mutations, outcomes, and the risk of recurrence of this manifold disease.

## 1. Introduction

Hemolytic uremic syndrome (HUS) is a rare disease characterized by thrombocytopenia, hemolytic anemia, and acute kidney injury, and is included in the group of thrombotic microangiopathies (TMAs). TMA is a histopathologic term used to describe a variety of thrombotic vascular lesions [1] leading to different pathologies depending on what organ is mainly affected and on the underlying pathophysiology. Kidneys are the main target organs of HUS, with fibrin and platelet thrombi in glomerular capillaries, endothelial cell swelling, detachment from the glomerular basement membrane, and consequent severe renal impairment [2].

The first reported HUS form was Shiga toxin-producing Escherichia Coli (STEC)-HUS, also known as “typical HUS” based on the association, described in the 1970s, between this gastroenteric infection and TMA [3].

In 2016, the International Hemolytic Uremic Syndrome provided the first classification of HUS into five groups [4]: 1. HUS with coexisting diseases or conditions (including hemopoietic stem cell transplantation, solid-organ transplantation, malignancy, autoimmune diseases, drugs, malignant hypertension, pre-existing nephropathy); 2. infection-induced HUS (consequent to S. pneumoniae, STEC, influenza A, H1N1, HIV, and, recently, SARS-CoV-2); 3. Cobalamin C defect–HUS; 4. DGKE–HUS (depending on a diacylglycerol kinase ε deficiency); 5. atypical HUS (aHUS). The latter is further split in HUS with a genetically determined dysregulation of the complement alternative pathway and HUS without an identified complement mutation or anti-CFH antibody.

In particular, genetic dysregulation of the alternative complement pathway, resulting in endothelial cell dysfunction and microvascular thrombi formation, can be identified in 40–60% of aHUS patients [5,6,7]. This dysregulation follows mutations in genes coding for complement regulatory proteins, such as factor H (CFH), factor I (CFI), membrane cofactor protein (MCP), complement 3 (C3), factor B (CFB), or thrombomodulin (THBD), or by the presence of anti-CFH antibodies with consequent hyperactivation of the complement system [8,9,10].

With the introduction of anti-C5 therapy (humanized monoclonal antibodies that block C5 activation), the natural history of aHUS completely changed, with a better prognosis, a rapid recovery of renal function, and a sharp reduction of end-stage kidney disease (ESKD) incidence in affected patients.

Today there is an increasing interest in the genetic bases of this severe disease. Indeed, an accurate and comprehensive genetic analysis is fundamental in the clinical routine to reinforce diagnosis and guide patient management and clinical decisions (as treatment with anti-C5 inhibitors). Moreover, it guarantees a correct assessment of the risk of recurrence, helping in establishing when to discontinue treatment and the consequent follow-up period. Finally, it is essential to correctly inform counseling and testing of at-risk relatives and to lead to an appropriate selection of kidney transplant recipients to ensure adequate pre- and post-transplant treatment.

The aim of this narrative review is to provide readers with a practical guide about different genes possibly involved, elucidating the specific role of each transcribed protein in the pathogenesis of aHUS. We focused on the main current evidence available in the literature about the relationship between genetic mutations, the outcome, and the risk of recurrence of this rare disease. For this purpose, an accurate literature search was performed. The PubMed database was searched on May 2023, using the following set of terms:

(((hemolytic uremic syndrome[MeSH Terms]) OR (HUS[Title/Abstract]) OR (aHUS[Title/Abstract]) OR (Hemolytic uremic syndrome[Title/Abstract])) AND ((Genetic*[MeSH Terms]) OR (Genome[MeSH Terms]) OR (Mutation*[MeSH Terms]) OR (Variant*[MeSH Terms]) OR (Gene*[MeSH Terms])))

All the original articles selected were published in the last 10 years, except for other additional articles identified and included by snowballing.

## 2. Defective Regulation of the Alternative Complement Pathway in aHUS

**Figure 1:**
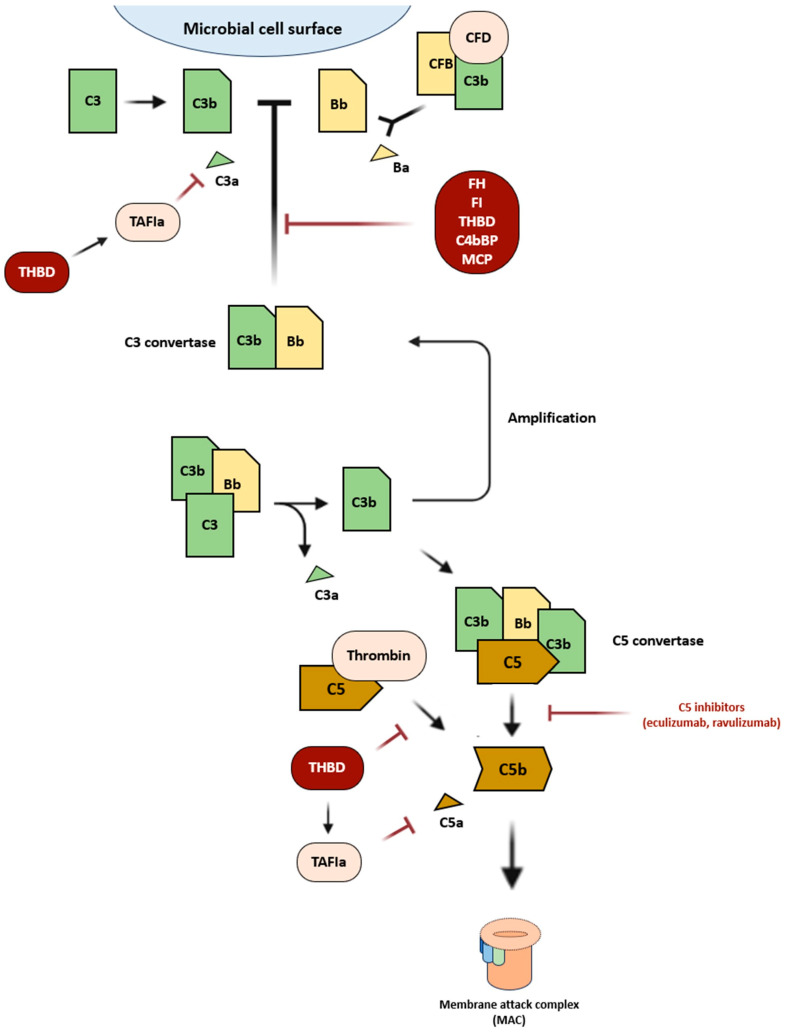
Activation of the alternative pathway of the complement system. CFB (complement factor B); CFD (complement factor D); TAFIa (thrombin activatable fibrinolysis inhibitor A); C4bBP (C4b binding protein); THBD (thrombomodulin); FH (complement factor H); FI (complement factor I); MCP (membrane cofactor protein).

The complement system is a major component of the innate immune system and is activated through three distinct pathways: classical, alternative, or lectin. They converge to form C3 convertase, a complex that primes membrane attack complex (MAC) formation. Particularly, the alternative pathway activates the innate immune system in the absence of antibodies [11] (Figure 1). C3b, upon encountering foreign elements, becomes activated and combines with factor B to create C3 convertase [12]. Thereafter, the C3 convertase attracts further C3b that, depositing on the cell membrane, promotes C5 convertase formation and, consequently, the MAC, whose function is to kill target cells [13]. This alternative route is controlled by several regulatory factors, such as THBD, CFH, and CFI [14,15]. Mutations of genes encoding for these regulatory factors lead to uncontrolled activation of the complement system, considered the main underlying mechanism of aHUS.

Genetically determined aHUS can be familial or sporadic. The familial form represents about 20% of cases and it is defined by at least two members of the same family being diagnosed with aHUS in a maximum period of 6 months [7]. Genetic mutations predisposing to aHUS can be inherited in either an autosomal dominant or autosomal recessive manner or, rarely, as polygenic inheritance [16]. 

Although several mutations are implicated in its pathogenesis, aHUS development is multifactorial. In fact, a second hit is necessary for the disease to manifest itself [17]: different triggers are associated such as drugs (cisplatin, gemcitabine, mitomycin, clopidogrel, quinine, interferon-alfa/beta, anti-vascular endothelial growth factor, alemtuzumab, cyclosporin tacrolimus, ciprofloxacin, oral contraceptives, and vaccines), infections (such as influenza, SARS-CoV-2), malignant hypertension, and pregnancy. Generally, mutations affect a single gene; however, the combination of two or more mutations has been described in about 12% of patients [18]. 

Among the principal involved genes (Table 1), we consider:

**Complement Factor H (*CFH*)**—The most frequently mutated gene (>30% of aHUS cases) encodes CFH and it is located on chromosome 1 (1q32) [7]. The *CFH* gene cluster is characterized by large repeated regions, favoring genomic rearrangements and copy number variations (CNVs) [19,20]. More than 120 *CFH* pathogenic or likely pathogenic variants have been identified in aHUS patients. Various mutations (missense, nonsense, small deletions/insertions) have been described, both in homozygosity and in heterozygosity, determining a quantitative (type I) or qualitative (type II) CFH deficiency [21]. CFH is a single-chain plasma glycoprotein of 150 KDa produced predominantly by the liver and made up of 20 structural domains called short consensus repeats (SCR), homologous to each other, consisting of about 60 amino acids [22,23]. It acts by competitively binding to C3b to inhibit the C3 convertase production, accelerating C3 convertase degradation, and as a cofactor for CFI in the cleavage of C3b [24]. Specifically, it interacts with various types of polyanions, such as heparan sulfates and salic acids, generating a greater affinity for C3b, with a consequent more effective inactivation [25]. Endothelial cells and the glomerular basement membrane are rich in polyanionic molecules, consequently being protected against an uncontrolled deposition of C3b on their surface [26]. According to different studies, mutated proteins show a low binding affinity both for the polyanions and for the C3b positioned on the endothelial cells' surface, determining a minor control of complement system activation [27]. Indeed, in most cases, mutations are not associated with low plasma levels of CFH, but only with an alteration of the cofactor activity. In several clinical studies, it has been observed that most patients with *CFH* mutations have clinically manifested the disease only in adulthood [28]. Moreover, genes encoding CFH and CFH-related (CHFR) proteins, located on the long arm of chromosome 1, are highly homologous and prone to non-allelic homologous recombination and conversion [29]. It results in deletions of *CFHR1*, *CFHR3*, or *CFHR4*, as well as the formation of hybrid genes such as *CFH-CFHR1* [30]. Some of these recombination or hybrid genes are associated with the production of CFH autoantibodies or malfunctioning hybrid proteins [31].

**Complement Factor I (*CFI*)**—Mutations of the *CFI* gene, located on chromosome 4 in the q25 region, are observed in 4–10% of aHUS cases. CFI is an 88 KDa plasma glycoprotein, mainly synthesized by the liver, characterized by an N-terminal heavy chain (H chain) and a C-terminal light chain (L chain), covalently linked by a disulfide bond [32]. The CFI regulates both the classical and the alternative pathways of the complement system by degrading C3b and C4b in a highly specific manner [33]. It needs several cofactors, including CFH, MCP, and C4bBP (C4b binding protein).

**Membrane Cofactor Protein (*MCP*)**—Mutations of the gene encoding MCP, located on chromosome 1 (1q32.2), are detected in 8–10% of cases. MCP is a transmembrane glycoprotein widely expressed on the surface of almost all human cell types, except erythrocytes, consisting of an N-terminal portion (composed of four SCRs), an STP region (serine, threonine, and proline), a hydrophobic transmembrane domain, and a C-terminal cytoplasmic tail [34]. It is a cofactor for CFI-mediated C3b and C4b cleavage. The four SCR regions bind C3b and/or C4b, facilitating their lysis by CFI. Homozygous or heterozygous mutations result in either reduced cell surface expression of MCP (type I mutation) or normal expression with reduced complement regulatory activity (type II mutation). According to several studies, *MCP* mutations predispose to aHUS [35]: indeed, the presence of mutant MCP on the cell surface and the potential exposure to triggering environmental factors could represent a risk factor for damage to the glomerular endothelial cells, with possible activation of the alternative pathway of the complement.

**Complement Factor B (*CFB*) and C3**—CFB and C3 mutations are uncommon. Their genes are located on chromosomes 6 (6p21.33) and 19 (19p13.3), respectively. *CFB* mutations result in an increased formation of C3 convertase with hyperactivation of the alternative complement pathway [36]. Instead, C3 is required for the formation of both C3 convertase and C5 convertase. Gain-of-function mutations of C3 result in C3b resistance to cleavage, prevention of inhibition by CFI, and raised affinity between C3b and CFB.

**Thrombomodulin (*THBD*)**—Heterozygous mutations of the *THBD* gene, encoding thrombomodulin, have been found in 3–4% of cases. The pathogenic mechanism is not fully understood yet. However, it seems to involve both the coagulation cascade and the complement system. As a matter of fact, thrombomodulin provides additional protection of the membrane by enhancing CFI-mediated inactivation of C3b in the presence of either CFH or C4bBP; by binding to thrombin, thereby preventing it from activating C5; and by promoting the generation from the thrombin activatable fibrinolysis inhibitor (TAFI) of an active carboxypeptidase, known as TAFIa, which inactivates C3a and C5a [37].

**Table 1:** Mechanism of action and mutation frequency of the main involved genes.

Gene	Locus	Mechanism of Action	Mutation Frequency
*CFH*	1q31.3	CFH competitively binds to C3b to inhibit the C3 convertase production, accelerating the C3 convertase degradation, and as a cofactor for CFI in the cleavage of C3b.	>30%
*MCP (CD46)*	1q32.2	MCP is a cofactor for CFI-mediated C3b and C4b cleavage.	8–10%
*CFI*	4q25	The CFI regulates both the classical and alternative pathways of the complement system by lysing C3b and C4b in a highly specific manner.	4–10%
*C3*	19p13.3	C3 is required for the formation of both the C3 convertase and C5 convertase.	2–10%
*CFB*	6p21.33	Combines with C3b to create C3 convertase.	0–3%
*THBD*	20p11.21	Encodes thrombomodulin, involved both in the coagulation cascade and the complement system.	3–4%

## 3. Genetic Mutations and Outcome: Is There a Connection?

Thanks to the development of different aHUS registries, collecting data from patients from several countries worldwide, it is now possible to determine thin connections between genetics and aHUS outcomes. Schaefer et al. [38], on behalf of the Global aHUS Registry, analyzed the correlation between genetic background and ESKD-free survival. They included in their analysis 851 patients (45% pediatrics, 55% adults), with a family history in 16% of them, finding complement system abnormalities in up to two-thirds of patients diagnosed with aHUS. Specifically, 119 patients (45%) reported a mutation in more than one aHUS-associated gene or anti-CFH: mutations in *MCP* turned out to be more frequent in childhood, while adult patients were more likely to have mutations of *CFI*. On the other hand, anti-CFH was common in both subgroups. They pointed out that age at first presentation was independent of the presence of any identified complement abnormality. Nonetheless, when patients with a single complement protein abnormality were analyzed individually, those with a *CFI* mutation were older at the initial presentation of aHUS than patients with other complement abnormalities in the overall population. At the same time, in the pediatric population, patients with anti-CFH antibodies presented at a significantly older age. Hence, anti-CFH antibodies proved to be the most commonly identified cause of aHUS in children aged 6 to 17 years.

ESKD-free survival at 1 and 5 years from initial disease presentation is clearly related to age at initial presentation, with more children than adults remaining ESKD-free after 5 years (73% and 51%, respectively). However, even if there were no differences among patients when analyzed by sex and the presence or absence of a complement abnormality, the authors underlined that patients carrying *CFH* mutations showed poor outcomes, with ESKD occurring more rapidly. On the other hand, patients with *MCP* mutations showed longer ESKD survival compared with those who tested negative for mutations in these genes. Other identified mutations, instead, did not affect time to ESKD, although numbers were limited for some genotypes. Additionally, outcomes for patients with anti-CFH antibodies were slightly inferior to those without anti-CFH antibodies. 

Finally, the authors also analyzed the extrarenal manifestations associated with aHUS. Reported rates resulted from 19% to 38% in recently diagnosed patients depending on the organ system. In particular, neurologic involvement was found in 24% of pediatric and 25% of adult patients with aHUS. The extrarenal involvement was evident in the chronic phase of the disease, demonstrating that all organs can continue to be affected following initial presentation. However, an assessment of the association of individual complement mutations with extrarenal manifestations was not possible due to the limited number of patients within each mutation group. Nonetheless, they underlined that, by continuing data collection, it may become possible to predict patients at risk of specific extrarenal complications through an accurate genetic assessment.

These data were strengthened by different authors. In particular, Piras et al. [39] reported the results of a retrospective study of *CFH-CFHR* copy number variation (CNV) analysis underlining the prevalent role of *CFH* genetic defects in the pathogenesis of aHUS, also proposing that abnormalities in CFHR proteins may play a role. The authors provided an in-depth characterization of resulting structural variants (SVs) in a large cohort of patients, including 258 patients with primary aHUS and 92 with secondary forms, evaluating the prognosis of patients carrying *CFH-CFHR* SVs and the contribution of the concomitant presence of rare complement gene variants or anti-CFH abnormalities to disease development. 

This study showed relevant associations between the specific SV and disease phenotype, response to therapies, and risk of recurrence after kidney transplant. Precisely, *CFH::CFHR1* hybrid genes were commonly found in patients whose disease onset is in their first year of life. Moreover, they pointed out that in the majority of cases, aHUS in *CFH::CFHR1* carriers was triggered by infections, suggesting that dysfunctional CFH could not adequately control the complement activation induced by the first exposure to pathogens in these patients. On the contrary, the reverse *CFHR1::CFH* hybrid genes were associated with a later aHUS onset. In any case, both hybrid *CFH::CFHR1* genes and reverse *CFHR1::CFH* genes in that cohort demonstrated a poor prognosis without anti-C5 therapy, confirming previous data. Nevertheless, they underlined that those treated with eculizumab reached a full remission. On the other hand, the outcome in patients with *CFHR* hybrids was better than in patients with *CFH* SVs, even if they did not receive C5 inhibitors, highlighting the lower pathogenic impact of *CFHR* SVs compared to *CFH* SVs. The authors also studied the association between *CFH* genetic abnormalities and the risk of relapses after kidney transplant, observing an adverse outcome in eight out of nine grafts without preventive eculizumab, while three grafts transplanted under eculizumab prophylaxis kept normal function.

Additionally, Merinero et al. [40,41] highlighted the poor prognosis associated with *CFH* gene mutations in patients with aHUS, stressing the importance of analyzing and characterizing *CFH* variants to guarantee a complete functional understanding of *CFH* genetic variability.

Particularly interesting is the study by Fujisawa et al. [42], who conducted a nationwide epidemiological survey of clinically diagnosed aHUS patients, examining 118 patients enrolled from 1998 to 2016 in Japan. They found out that the most frequent genetic abnormalities were in *C3* (31%), while the frequency of *CFH* variants was relatively low (10%), totally different compared to Western countries. 

The authors compared the clinical characteristics of aHUS patients according to genetic or acquired backgrounds. They observed that the initial episode usually occurred during childhood in the *C3*, *MCP*, and unidentified groups, while patients with anti-CFH antibodies showed a biphasic distribution of age at aHUS onset (childhood in 80% and middle-to-old age in the remaining 20%). Severe anemia was observed in patients with *CFH* variants (38%), while C3 levels varied depending on complement abnormalities: indeed, half of the patients with *C3* and *MCP* variants and 89% of the patients with anti-CFH antibodies showed decreased levels of C3. No difference could be observed in the mortality rate in the acute phase among different mutations. Nevertheless, at discharge, the overall renal mortality rate was higher in *CFH* variants (38%) and the unidentified group (24%). Similarly, the risk of death or ESKD in the acute phase did not differ by treatment choice for any mutated genes. Patients were, then, followed for a median of 2.5 years: relapses occurred in patients with *C3* (77%), *MCP* (50%), and *CFH* (38%) variants. All three patients who had renal transplantation experienced aHUS relapse, and the one with the *C3* variant redeveloped ESKD, while the other two (one with a *CFH* variant and the other in the unidentified group) recovered renal function.

Overall, a higher frequency of *C3* variants and anti-CFH antibodies and a lower frequency of *CFH* variants are associated with a better prognosis of Japanese aHUS (with a total mortality rate of 5.4% and a renal mortality rate of 15%, as reported by the authors), drawing attention to genetic testing and reinforcing the available data on the poorest prognosis in patients with *CFH* variants.

## 4. C5 Inhibitors Discontinuation: Is There a Path to Follow?

Considering the essential role of anti-C5 therapy to prevent aHUS relapses, patients diagnosed with aHUS usually start a lifelong treatment with eculizumab every two weeks, with a consequent reduction of their compliance in the long run, entailing significant health costs. Even if the modern ravulizumab clearly improved the management and quality of life of aHUS patients, and reduced the economic impact of this therapy, our primary goal should be to safely suspend treatment, and if possible, monitor patients in an outpatient setting. Nevertheless, the main obstacle is to find a guiding criterion to decide the correct timing for therapy discontinuation.

Fakhouri et al. [43] report data from a large cohort with aHUS, in whom eculizumab was discontinued after a period of treatment of at least 6 months. The authors demonstrated that complement genetic variants do not affect the response to eculizumab. However, they are major predictive factors for aHUS relapse after eculizumab discontinuation. The risk of relapse after therapy interruption was the highest in patients with *CFH* variants (72%), reflecting the severity of the disease in these patients. Those with *MCP* variants represent the second group of patients with an equally relatively high risk of relapse after eculizumab discontinuation (about 50%). Compared with *CFH* variant carriers, relapses in patients with *MCP* variants tended to occur later after therapy discontinuation (> 1 year), but the median time to relapse after eculizumab stop did not significantly differ between patients with *CFH* variants and those with *MCP* variants. Interestingly, none of the patients with no detected complement gene variants had aHUS relapse after a relatively long median follow-up (17 months). However, their series did not provide sufficient data for patients with *C3*, *CFI*, or *CFB* variants or anti-CFH antibodies. Nevertheless, they underlined that a titer of anti-CFH antibodies below a pathogenic threshold of 1000 IU/mL may allow treatment suspension with anti-CFH antibody monitoring. 

Altogether, from these data, eculizumab discontinuation seems to be safe in patients with no documented complement gene variants after 6–12 months of treatment. In contrast, patients with *CFH* variants and patients with *MCP* variants have a high risk of relapse after eculizumab discontinuation.

The same group, in a more recent study [44], supported its conclusions. Indeed, they observed that aHUS relapse risk after eculizumab discontinuation is predominantly determined by the presence or the absence of a rare variant in a complement gene. In particular, the absence of a complement gene variant proved to be the most powerful negative predictive factor (no relapses occurring in 26 patients with aHUS without a complement gene variant); on the other hand, relapses occurred in 12 of 28 patients with a complement gene variant (with a positive predictive value of 43%; 95% CI: 25–61%). The risk was the highest (about 50%) in carriers of variants in *CFH* and *MCP* genes, and the lowest (<5%) in patients without detected variants. Therefore, in a patient with no detected variant, aHUS relapse should lead to a reassessment of genetic results or even of aHUS diagnosis. 

Moreover, several features were identified, in addition to complement genetics, to help in decision making. First of all, female patients seemed to be at increased risk of relapse compared with male patients. An elevated sC5b-9 level at therapy interruption was independently associated with a risk of relapse. 

In both studies, all patients who experienced a relapse rapidly resumed eculizumab, resolving AKI and thrombocytopenia and recovering baseline renal function. Furthermore, the authors stressed that close patient monitoring is fundamental to guarantee an early restart of treatment in the case of recurrence.

Brodsky, in [45], underlined that this study increased our confidence about eculizumab discontinuation in most patients with rare germline complement mutations, even if it was not clear whether this holds for patients with factor H autoantibodies, considering that there were too few patients. Moreover, another limitation of the study is that germline mutations of *CFH* are usually the most common mutations, but not in this study (MCP resulted mutated in 43% of cases). Furthermore, the study included nine patients (16%) who experienced more than one episode of aHUS before study entry, potentially biasing the study and overestimating the relapse rate. In any case, the author highlighted that patients carrying germline variants, considered at higher risk, have a relapse rate of 50% or less, wondering if it is appropriate to also offer them a trial of C5-inhibitor discontinuation under careful supervision. Finally, the mean follow-up was less than 18 months. This short period of observation significantly limits the conclusion of the study. Hence, the author asked for further prospective studies.

Similarly, Bouwmeester et al. [46] further discussed this nonrandomized study's limitations. The authors first underlined a clear bias in patient selection: eculizumab withdrawal was attempted in only 56% of patients (30 of 52) who were newly diagnosed with aHUS at enrolling sites during the study period. Moreover, the study cohort included only six patients (11%) with a *CFH* mutation, even if *CFH* gene mutations are more common in most aHUS cohorts, as previously reported, suggesting that there was probably a hesitance in attempting withdrawal in these individuals. Furthermore, we have no information about other relevant clinical parameters that might have affected the decision of discontinuation (such as blood pressure control, cardiovascular diseases, patient compliance, etc.) and about therapy interruption in kidney transplant recipients (who are more at risk of having a relapse and less likely to recover baseline GFR). Finally, the optimal timing and the role of soluble C5b-9 in routine clinical practice are not clearly defined. Nevertheless, the authors confirmed the utility of this study in demonstrating that eculizumab withdrawal is feasible in some patients with aHUS, although the relapse rate was not negligible among patients with complement gene variants. These findings strengthened the necessity of full genetic evaluation in aHUS patients and of further studies on this topic.

Acosta-Medina et al. [47] attempted to estimate the role of complement genetic variants in aHUS recurrence after the discontinuation of C5 inhibitors, analyzing data from 280 patients from 40 different studies. Variants were identified in 60% of patients (most commonly *CFH* and *MCP/CD46*). Half (51.3%) of them had ≥1 likely pathogenic/pathogenic variant, whereas the remaining had variants of uncertain significance (VUS). The overall relapse rate after therapy discontinuation resulted to be 29.6%. Complement genetic variants were associated with a three-fold increase in the risk of relapse after therapy withdrawal, without differences among individuals with single or multiple concomitant variants. *CFH* and *C3* variants were associated with the highest risk of relapse, particularly the variants in *CFH* exon 22. Moreover, a prominent and significant increase in relapse risk was also observed among patients with variants in the *MCP/CD46* gene. However, even if the highest risk was demonstrated in the population with pathogenic/likely pathogenic variants, they provided proof of a relevant risk of relapse in patients with VUS. Therefore, until these genetic alterations are better characterized, continuation of eculizumab may be recommended. 

On these bases, the authors provided a possible framework regarding how complement genetics (together with patient-specific factors, such as age, baseline renal function, and prior kidney transplant) can guide eculizumab discontinuation. Indeed, they suggested that eculizumab discontinuation should be considered after at least 6 months of treatment with a complete hematologic response and a stable renal function for at least 3 months. 

If aHUS occurs in patients < 18 years at the time of first symptoms or in patients with kidney graft, they warned to perform a complete complement gene panel and antibody evaluation (including *CFH*, *MCP/CD46*, *CFI*, *C3*, *CFB*, *THBD*, ADAMTS13, anti-CFH, and *CFHR1-5*) and, in case of:

- ≥1 variant reported as pathogenic/likely pathogenic in *CFH*, *MCP/CD46*, *C3*, *CFI*, and *CFB*;
- positive anti-CFH titer despite immunosuppressive treatment;
- ≥1 VUS in *CFH*, *C3*, or splice region variant in *MCP/CD46*;

longer-term or lifelong therapy should be considered.

In any case, as in the previous studies, these authors also highlighted that is crucial to strictly monitor patients once therapy is discontinued to guarantee C5 inhibitors as soon as a relapse is detected.

Recently, Bouwmeester et al. [48] reported the results of the CUREiHUS study, a 4-year prospective, observational study monitoring unbiased eculizumab discontinuation in Dutch patients with aHUS after 3 months of therapy. A total of 21 patients, both pediatrics and adults with aHUS in native kidneys and a first-time eculizumab treatment were included. In 17 patients (81%), a defect in complement regulation was found by either a proven or likely pathogenic mutation or a VUS in one of the complement regulatory genes or by antibodies against factor H. All patients showed full recovery of hematological TMA parameters after the start of C5-inhibitor and a renal response was noted in 18 patients. After a median treatment duration of 13.6 weeks, eculizumab was withdrawn in all patients. 

During a follow-up of 80.7 weeks after treatment discontinuation, the relapse rate was 22% (4 patients), with a median time to first relapse of 19.5 weeks. All relapses were detected early, with no need for dialysis; moreover, no extrarenal manifestations of TMA were observed. The C5 inhibitor was promptly restarted in all relapsing patients (within 24 h), and no chronic sequelae and/or hypertension was observed in relapsing patients at the last follow-up. Even if the authors underlined that the low sample size and event rate did not allow them to determine predictors of relapse, they stressed that relapses only occurred in patients with a likely pathogenic variant.

Furthermore, the study also provides an extensive cost/consequence analysis, showing that eculizumab withdrawal reduced the medical costs per patient by 70% without negatively affecting quality of life.

Therefore, this study further highlighted that an early treatment withdrawal (median 3 months) is safe, feasible, and cost-effective, but larger data registries are needed to determine factors to predict relapses and long-term outcomes of eculizumab discontinuation.

Indeed, Noris et al. [49], in their comment on this study, underlined that in the high-risk group of aHUS patients with pathogenic or likely pathogenic complement gene variants, 50% to 75% of patients did not relapse. Hence, it is not clear yet if we may offer high-risk patients the opportunity to discontinue eculizumab and how it is possible safely, considering the current absence of early and reliable predictors of aHUS relapse.

In addition, the authors stressed the necessity to further investigate the long-term effects associated with a chronic activation of complement even in the absence of distinct clinical signs of relapse.

## 5. Conclusions

From this narrative review, it seems clear that, even if aHUS is well known today, different questions are still unsolved, representing an important and promising field of research. A full knowledge of all the underlying molecular and genetic mechanisms is essential to guarantee steps forward in the treatment of this rare disease and in the management of these frail patients.

Considering its rarity, the only way to ensure progress in this area of interest is to stimulate all specialized centers to undergo routine genetic screening of all their patients in order to increase the amount of data about the influence of complement abnormalities on disease characteristics and progression. In particular, it emerges that further studies are necessary to understand the meaning of numerous VUS, not only of *CFH* and *MCP*, but also of other involved genes (such as *CFI*, *C3*, *CFB*, and *THBD*). Indeed, it may be useful to better understand all the slight connections among different mutations and specific aHUS phenotypes.

Moreover, despite several limitations, Fakhouri et al. [43,44], Acosta-Medina et al. [47], and Bouwmeester et al. [48] gave a spark of hope for a possible safe therapy discontinuation through an accurate genetic evaluation of the single patient. 

Nevertheless, more studies are necessary to accurately determine genetic predictors of aHUS relapses and to guarantee a safe therapy withdrawal. Further research is needed to identify predictive biomarkers and patient features that may help in treatment tapering and discontinuation. 

However, even if lifelong therapy is currently recommended in the present regulatory guidance, we have a social responsibility to ensure a feasible, safe, and controlled eculizumab withdrawal in patients with aHUS in the next years in order to improve their quality of life and to reduce the huge costs for the national health system.

## Data Availability

Data sharing not applicable.
